# Supplementary material for: Very long-chain fatty acids drive 1-deoxySphingolipid toxicity
Source: Nat Commun. 2025 Nov 26;16:11650. doi: 10.1038/s41467-025-66687-8 (PMC12749609; doi:10.1038/s41467-025-66687-8)
Supplement: Supplementary file 1 — Supplementary Information [file 41467_2025_66687_MOESM1_ESM.pdf]

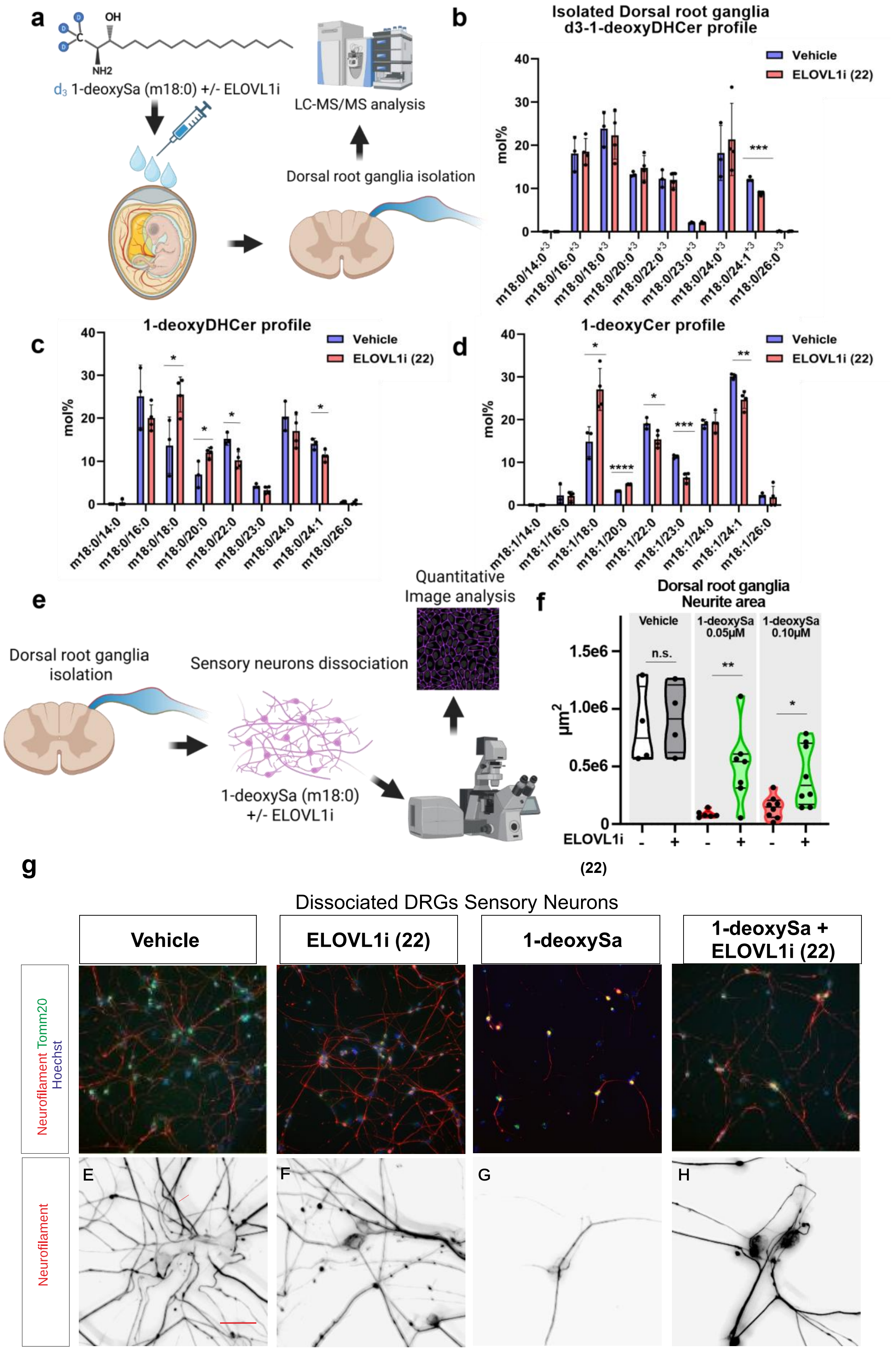

a
OH
NH2
d3 1-deoxySa (m18:0) +/- ELOVL1i
LC-MS/MS analysis
Dorsal root ganglia isolation
b
Isolated Dorsal root ganglia
d3-1-deoxyDHCer profile
Vehicle
ELOVL1i (22)
mol%
m18:0/14:0+3
m18:0/16:0+3
m18:0/18:0+3
m18:0/20:0+3
m18:0/22:0+3
m18:0/23:0+3
m18:0/24:0+3
m18:0/24:1+3
m18:0/26:0+3
c
1-deoxyDHCer profile
Vehicle
ELOVL1i (22)
mol%
m18:0/14:0
m18:0/16:0
m18:0/18:0
m18:0/20:0
m18:0/22:0
m18:0/23:0
m18:0/24:0
m18:0/24:1
m18:0/26:0
d
1-deoxyCer profile
Vehicle
ELOVL1i (22)
mol%
m18:1/14:0
m18:1/16:0
m18:1/18:0
m18:1/20:0
m18:1/22:0
m18:1/23:0
m18:1/24:0
m18:1/24:1
m18:1/26:0
e
Dorsal root ganglia isolation
Sensory neurons dissociation
Quantitative Image analysis
1-deoxySa (m18:0) +/- ELOVL1i
f
Dorsal root ganglia
Neurite area
Vehicle
1-deoxySa 0.05µM
1-deoxySa 0.10µM
n.s.
µm2
1.5e6
1.0e6
0.5e6
ELOVL1i
(22)
g
Dissociated DRGs Sensory Neurons
Vehicle
ELOVL1i (22)
1-deoxySa
1-deoxySa + ELOVL1i (22)
Neurofilament Tomm20
Hoechst
Neurofilament
E
F
G
H

Supplementary Figure 1 Pharmacological inhibition of ELOVL1 mitigates 1-deoxysphingolipids induced neuronal toxicity in dorsal root ganglia

1 a Schematic overview of the in vivo d_3-1-deoxySa (m18:0) flux assay using whole chicken embryos. Embryos were supplemented with d_3-1-deoxySa (m18:0), followed by isolation of dorsal root ganglia (DRG) and high-resolution LC-MS/MS lipidomics analysis. **b, c, d** Quantification of d3-1-deoxyDHCer (m18:0/xx:y^{+3}), 1-deoxyDHCer (m18:0/xx:y) and 1-deoxyCer (m18:1/xx:y) species in DRG after the whole chicken embryo supplementation with d_3-1-deoxySa in presence (n = 4, individual chicken embryos) or absence (n = 3) of the ELOVL1 I inhibitor (22) treatment. Data were normalized to internal standards and sum of all labeled d3-1-deoxyDHCer, 1-deoxyDHCer, 1-deoxyCer respectively. **e** Schematic of the DRG neurotoxicity assay. DRGs were isolated from 9-day-old chicken embryos, dissociated, and cultured with 1-deoxySa in the presence or absence of the ELOVL1 inhibitor (22). Neuronal toxicity was assessed via fluorescence microscopy and quantitative image analysis. **f** Quantification of neurite area in dissociated DRG neurons treated with 1-deoxySa (0.05 µM or 0.1 µM) or vehicle (DMSO), in the presence or absence of ELOVL1i (22). Neurites were labeled with anti-Neurofilament-M, mitochondria with anti-TOMM20, and nuclei with DAPI. Imaging was performed at 20× magnification, and total neurite area was quantified using ImageJ. Each data point represents a total neurite area of the entire well. **g** Representative fluorescence images of DRG neurons treated with vehicle or 1-deoxySa (0.05 µM) ± ELOVL1i (22). Panels **A–D** show axonal networks at 20× magnification; panels **E–H** show close up images along axons. Scale bars: 100 µm (**A–D**); 25 µm (**E–H**). Significance was determined using two-tailed unpaired Student’s t-test. $p < 0.05$ (*), $p < 0.01$ (**), $p < 0.001$(***), $p < 0.0001$(****). Illustrations were created in BioRender. Hornemann, T. (2025) https://BioRender.com/ifocfkk

Supplemental Table 1

	NH					NP			PEP		TH	NoN
	NF1	NF2/3		NF4/5		NP1	NP2/3		PEP1	PEP2		
		NF2	NF3	NF4	NF5		NP2	NP3				
Elovl1	0.097	0.063	0.000	0.000	0.038	0.272	0.281	0.250	0.063	0.118	0.253	0.027
Elovl2	0.000	0.021	0.000	0.000	0.000	0.008	0.000	0.000	0.000	0.000	0.116	0.014
Elovl3	0.000	0.000	0.000	0.045	0.000	0.000	0.000	0.000	0.000	0.000	0.000	0.000
Elovl4	0.516	0.625	0.833	0.364	0.731	0.328	0.250	0.250	0.063	0.059	0.215	0.027
Elovl5	0.097	0.417	0.167	0.409	0.577	0.112	0.094	0.250	0.281	0.176	0.107	0.095
Elovl6	0.290	0.063	0.250	0.045	0.000	0.344	0.313	0.000	0.125	0.059	0.197	0.041
Elovl7	0.742	0.708	1.000	0.227	0.154	0.400	0.313	0.417	0.188	0.412	0.515	0.041
Cers1	0.355	0.354	0.583	0.182	0.308	0.016	0.063	0.083	0.063	0.294	0.073	0.027
Cers2	0.000	0.000	0.000	0.000	0.000	0.176	0.156	0.250	0.109	0.000	0.073	0.027
Cers3	0.000	0.000	0.083	0.000	0.000	0.016	0.031	0.000	0.000	0.000	0.000	0.000
Cers4	0.129	0.021	0.000	0.000	0.038	0.280	0.094	0.000	0.203	0.059	0.262	0.014
Cers5	0.097	0.042	0.083	0.136	0.077	0.144	0.094	0.000	0.266	0.059	0.116	0.027
Cers6	0.032	0.083	0.000	0.045	0.077	0.000	0.031	0.000	0.000	0.059	0.017	0.000
Scn10a	0.000	0.000	0.000	0.000	0.000	0.760	0.625	0.417	0.281	0.588	0.227	0.014
Trpa1	0.000	0.000	0.000	0.000	0.000	0.512	0.219	0.167	0.063	0.000	0.176	0.014
Trpv1	0.000	0.000	0.000	0.045	0.000	0.032	0.281	0.583	0.313	0.059	0.000	0.000
Ntrk2	0.387	0.104	0.000	0.000	0.000	0.000	0.000	0.000	0.016	0.000	0.013	0.081
Ntrk3	0.161	0.250	1.000	0.545	0.731	0.104	0.063	0.000	0.031	0.176	0.300	0.041
Actb	0.903	0.708	0.500	0.591	0.577	0.744	0.656	0.750	0.688	0.882	0.794	0.432
Co-expression	0.000	0.000	0.000	0.000	0.000	0.064	0.031	0.167	0.000	0.000	0.017	0.000

Supplemental Table 1 Gene expression profiles in dorsal root ganglion cells. *CERS2* and *ELOVL1* are co-expressed in nociceptors related to pain and temperature perception. DRG neurons classified based on single cell RNA-seq can be divided into 11 types (*NF1-5, NP1-3, PEP1-2, and TH*) 28. A co-expression of *ELOVL1* and *CERS2* is primarily seen in the neuron classes *NP1-3*, which express pain receptors (*Scn10a, Trpa1, Trpv1*) and in the TH subtype, which are involved in mechanical pain. No strong *ELOVL1* and *CERS2* co-expression is seen in other neuron types such as low threshold mechanoreceptors or proprioceptive neurons, nor in non-neuronal cell types. Other *ELOVL* and *CERS* isoforms are either ubiquitously expressed in all neuron subtypes, or not expressed at all. Thus, the distinctive *ELOVL1* and *CERS2* co-expression in a specific subset of DRG neurons potentially contributes to tissue selectivity.
